# Corneal Ulcerations

**Published:** 1908-09-26

**Authors:** Philip A. Harry


### OPHTHALMOLOGY.

### CORNEAL ULCERATIONS.

By PHILIP A. HARRY, M.D.

From the point of view of general medical practice, corneal ulcers may be conveniently divided into two classes, traumatic and constitutional.

The former are usually associated with some more or less definite history of injury, *e.g.* an embedded foreign body, a scratch, a burn, anything causing an abrasion of the corneal epithelium, followed by infection. Even in cases of purulent or muco-purulent inflammation of the conjunctiva, it requires some injury to remove this protective anterior epithelium before infection will occur.

This class of ulcer is usually situated in the lower half or lower three-fourths of the cornea, that part of it left exposed by the palpebral fissure. An exception to this might be mentioned. A sharp particle of sand, stone, or coal may strike the unprotected cornea with sufficient force to be temporarily embedded in the epithelium. In the reflex act of winking the upper lid passes over the foreign body, dislodges it, and sweeps it into the upper cul-de-sac; there it may be found when the lid is everted, on its posterior surface, about the middle. Each act of winking causes the sharp edges of the foreign body to rub against the corneal epithelium, producing abrasions. Each abrasion exposes a fresh set of sensitive nerve endings. Eventually the patient is compelled to keep both eyes closed, or else suffer indescribable agony.

If the cornea, in a case like this, be suitably stained, and examined with Berger's binocular loupe, numerous streaks will be discovered in the upper part of the cornea. It is interesting to note that these streaks are more often horizontal or oblique rather than, as might be expected, vertical. Infection leads to a large superficial ulcer in the upper half of the cornea. The epithelium, however, soon regenerates, and the risk of infection is slight.

The constitutional ulcer may be situated in any part of the cornea. More often than not it is situated exactly in the centre—the part of the cornea furthest away from the blood supply. This is especially the case in weakly, tuberculous children, in whom the condition is also nearly always bilateral; and in old people whose corneæ are badly nourished. Ulcerations that may be classed as constitutional include the marginal, gouty, or rheumatic ulcer, the phlyctenular ulcer, the malarial ulcer, the herpetic ulcer, etc. These ulcerations are longer in healing than the traumatic, and depend less on local than on general treatment. Both types, and especially the constitutional, are liable to infection by the various organisms found on the conjunctiva. Infection is shown by the blurred, ill-defined margins and the steaminess of the cornea in the immediate vicinity. The actually ulcerated area may be readily shown up by a drop of the following stain: 2 per cent. fluorescein mixed with a 3 per cent. borax solution.

The important point to remember is that whereas traumatic ulcerations may be treated with irritating antiseptics, the constitutional ulcers will not improve under such treatment. They require soothing, non-irritating applications—normal saline or zinc lotion (1 in 500), instead of perchloride of mercury or formalin lotions.

Continual re-infection will keep an ulcer of the second type active for some time. The side nearest the corneo-scleral margin or limbus may be quite healed with a sheaf of blood-vessels growing towards it, while the side furthest away shows no sign of healing. This is often noticed in the upper part of the cornea in phlyctenular ulcerations.

Traumatic ulcers which have been infected with virulent organisms, usually in patients with disease of the lachrymal apparatus, ozæna, etc., will often eat their way across the cornea from one side to the other, or from periphery to centre. The insidious yet certain way in which they do this has given rise to the name serpent or spreading ulcer. Such an ulcer is usually noticed in the lower half of the cornea, and is very often associated with a collection of pus in the anterior chamber and with inflammation of the iris, from the vessels of which pus cells escape.

A few ulcers grow directly backwards into the substance of the cornea and threaten perforation. The "harvest" ulcer is of this nature. Such cases should be treated vigorously; the ulcer should be well cauterised with the actual or the galvano-cautery. If necessary, the lachrymal sac should be opened into by way of the canaliculus and syringed out with some strong antiseptic lotion. Stimulating antiseptic drops should be used—peroxide of hydrogen, chlorine water, alcohol, etc. The pupil must be kept dilated. Local pain can be relieved by warm fomentations or by local bleeding. The constitutional type needs general tonic treatment, fresh air, nourishing food, and a regular mode of life, with the addition of stimulants for the aged.

